# *ENPP1* deletion causes mouse osteoporosis via the MKK3/p38 MAPK/PCNA signaling pathway

**DOI:** 10.1186/s13018-022-03349-1

**Published:** 2022-10-15

**Authors:** Qiang Wang, Zhiqiang Gao, Kai Guo, Jiawei Lu, Feng Wang, Yufeng Huang, Desheng Wu

**Affiliations:** Department of Spine Surgery, Shanghai East Hospital, Tongji University School of Medicine, 150 Jimo Rd., Shanghai, 200120 China

**Keywords:** Osteoporosis, Proliferation, Enpp1, p38 MAPK, Lysophosphatidic acid

## Abstract

**Background:**

Apart from the current understanding of enzyme function, the mechanism of ectonucleotide pyrophosphatase/phosphodiesterase 1 (*Enpp1*) deficiency-associated osteoporosis is unknown. We aimed to explore the changes in the expression of signaling pathways of bone tissues involved in *Enpp1* deficiency.

**Methods:**

The body weights and morphology and histology of the bones of male *Enpp1* knockout (KO) and wild-type (WT) mice were assessed. The humeri of WT and *Enpp1* KO mice at 12 weeks of age were subjected to high-throughput quantitative molecular measurements, and bioinformatics analysis was performed. Proteins from humeri and calvarial pre-osteoblasts (Pobs) were used to verify the differentially expressed signaling pathways and to explain the mechanism of *Enpp1* deficiency-associated osteoporosis.

**Results:**

*Enpp1* KO mice had significantly lower body weight and trabecular bone mass in the hindlimbs than WT mice. Proteomics and immunoblotting showed that *Enpp1* deletion downregulated the expression of the p38 mitogen-activated protein kinase (MAPK) signaling pathway in bones. Lysophosphatidic acid (LPA) was involved in activating the MKK3/p38 MAPK/PCNA pathway and proliferating Pobs in *Enpp1* KO mice, whereas a p38 MAPK inhibitor suppressed the LPA-induced pro-proliferation phenotype (*p* < 0.05).

**Conclusion:**

The inhibition of MKK3/p38 MAPK/PCNA pathway plays an important role in the development of osteoporosis caused by *Enpp1* deficiency, and LPA partially rescued the proliferation of pre-osteoblasts via the MKK3/p38 MAPK/PCNA pathway.

**Supplementary Information:**

The online version contains supplementary material available at 10.1186/s13018-022-03349-1.

## Introduction

Ectonucleotide pyrophosphatase/phosphodiesterase 1 (*Enpp1*), also known as plasma cell membrane glycoprotein 1 (PC-1), is highly expressed in a variety of tissues, including bone, cartilage, and adipose tissues, and has highlighted its importance in human health and disease [1, 2]. *Enpp1* is a central regulator of bone development in mammals [3], where it catalyzes the synthesis of inorganic pyrophosphate (PPi), which inhibits hydroxyapatite (HA) precipitation, thus preventing over-mineralization [4]. Humans with *Enpp1* deficiency exhibit bone mineralization disturbances, resulting in early-onset osteoporosis [5]. Altered mineralization by defective *Enpp1* expression was initially demonstrated using the “tiptoe walking” (ttw/ttw) mouse model [6]. Nucleotide disruption in exon 9 of the *Enpp1* gene exhibited abnormalities that were almost identical to those present in the ttw/ttw mice, including decreased levels of extracellular PPi and substantial alterations in the mineralization of long bones and calvariae [7]. However, the molecular mechanism by which *Enpp1* loss leads to osteoporosis remains unclear. We aimed to explore the changes in the signaling pathway expression of bone tissues involved in *Enpp1* deficiency and reverse the phenotype of osteoporosis caused by *Enpp1* deletion, and it will provide reference significance for future clinical work.

## Materials and methods

### Experimental animals and analysis of skeletal phenotype

This study was in strict accordance with the recommendations of the 2018 “Guide for the Care and Use of Laboratory Animals” (NIH, Bethesda, MD). All procedures involving animals and their care in this study were reviewed and approved by the ethical review board of the Shanghai East Hospital of Tongji University (Ethical approval code: EC. D (BG). 016.02.1). The technical routes are as follows: The Enpp1 has 5 transcripts; according to the structure of Enpp1 gene, exon2 of Enpp1-203(ENSMUST00000135846.1) transcripts is recommended as the knockout region contains 73 bp coding sequence. CRISPR/Cas9 technology was used to modify Enpp1 gene. The brief process is as follows: sgRNA was transcribed in vitro. Cas9 and sgRNA were microinjected into the fertilized eggs of C57BL/6J mice, and fertilized eggs were transplanted to obtain positive F0 mice which were confirmed by PCR and sequencing. A stable F1 generation mouse model (heterozygous mice) was obtained by mating positive F0 generation mice with C57BL/6J mice. Both wild-type (WT) and KO mice were bred from heterozygous mice. Genotyping was performed on genomic DNA isolated from the toes and analyzed using PCR protocols developed by GeneTyper (GeneTyper, New York, USA). The mice were maintained in cages under controlled environmental conditions (22 ± 2 °C, 55–65% humidity, and a light/dark cycle of 12/12 h). Normal chow diet and freshwater were provided during the experimental period. The female and male mice exhibited similar phenotypes, and the phenotype of male mice was shown in article. The body weights of all mice were measured weekly. The tiptoe walking posture and body flexibility of the mice in the same age and sex groups were observed regularly. Skeleton staining was performed as described by Shull [8].

### Cell culture

Primary pre-osteoblasts were isolated by dissecting the calvaria from euthanized neonates (2–3 days old), as mentioned previously [9]. The obtained calvarial pre-osteoblasts (Pobs) were isolated and maintained in a regular growth medium containing α-MEM supplemented with 10% FBS at 37 °C in a 5% CO_2_-humidified incubator. Half media changes were performed on every third day. Prior to lysophosphatidic acid (LPA, an activator of MAPK signaling pathway) and SB203580 (inhibitor of p38 MAPK) stimulation, the cells were serum-deprived by incubating in α-MEM containing 0.1% essential fatty acid-free BSA for 24 h.

### Micro-computed tomography (µCT) analysis

Left hindlimbs of 12-week-old mice were harvested and soaked in neutral formalin. Bone morphology and microarchitecture were assessed at the distal femoral metaphysis for trabecular parameters. Femurs were scanned with 2 K resolution, 10-μm voxel size, 0.5 Al filter at 60 kV, and 167 μA. Trabecular bone in the 1000 μm above the growth plate and extending proximally for 1000 μm was measured for analysis. Images were reconstructed using NRecon version 1.1.11 (Bruker micro-CT) and analyzed using CTAn, v1.15 (SkyScan1176 in vivo micro-CT; Bruker).

### Hematoxylin and eosin (HE) and saffron solid green staining and immunohistochemistry

HE and saffron solid green staining and immunohistochemistry were performed as previously described [10, 11]; harvested right hindlimbs were decalcified and embedded in paraffin blocks, and each section was deparaffinized, rehydrated, and stained with HE, saffron solid green, and immunohistochemical staining. The tissue slices were incubated with Ki67 (nuclear proliferation-associated antigen Ki67,1:100; product code ab15580; Abcam), PCNA (proliferating cell nuclear antigen, 1:100; product code ab29; Abcam), RUNX2 (Runt-related transcription factor 2,1:100; product code ab92336; Abcam), and OCN (osteocalcin,1:100; product code ab93876; Abcam) and then incubated with HRP-conjugated secondary antibodies. The results were captured by fluorescence microscopy and quantified using Image-Pro Plus 6.0 (Media Cybernetics), and the mean density value (integrated optical density divided by the relevant area) was calculated for each visual field.

### Immunofluorescence staining

Immunofluorescence was performed according to the protocol described by Willemsen [12], followed by incubation with primary antibody against Enpp1 (1:1000, Bioss Antibodies Inc., Woburn, MA, USA) and secondary antibody (Alexa Fluor 488 Labeled Goat Anti-Rabbit IgG, Beyotime, product code A0423). TdT-mediated dUTP-biotin nick end labeling (TUNEL) staining was performed as previously described by Markaryan [13]. For EdU labeling in vivo, EdU (50 mg/kg body weight) was injected intraperitoneally into mice daily for seven consecutive days, and the hindlimbs were removed and processed for EdU immunostaining after decalcification. The pulse and chase time periods were chosen based on previous reports [14]. Proliferation and apoptosis index were calculated as the percentage of positive cells observed under the fluorescence microscope and were quantified using Image-Pro Plus 6.0 (Media Cybernetics).

### High-throughput quantitative molecular measurement

Four male WT mice and four *Enpp1* KO mice at 12 weeks of age were euthanized. Humeri were separated, and bone marrow in humeri was removed by 120,000 × g high-speed centrifugation. Freshly frozen humeri tissues from four biological replicates of each group (a total of eight mice) were processed. Bone protein was prepared from humeri, and protein identification and quantitative analysis using Nano-UPLCMSE tandem mass spectrometry were performed as described by Yang [15]. Heatmaps related to signaling pathways were generated using heatmap function of R. Gene Ontology (GO) functional annotations and Kyoto Encyclopedia of Genes and Genomes (KEGG) pathways were obtained using R Studio. The MCODE cluster analytical tool was used to generate protein–protein interaction networks.

### CCK-8 assay

CCK-8 kit (Dojindo, Shanghai, China) was used to measure the cell proliferation. A total of 1000 cells in a volume of 100 μL per well were cultured in a medium containing 10% FBS, in eight replicate wells in a 96-well plate. Then, the CCK-8 reagent (10 μL) was added to 90 μL DMEM to generate a working solution, of which 100 μL was added per well and incubated for 4 h. The OD at 450 ƞm was recorded for the analysis.

### Western blotting

Total protein was extracted from humeri and Pobs of WT and KO mice using RIPA lysis buffer supplemented with protease and phosphatase inhibitors. Equal quantities of protein (40 µg per lane) were subjected to SDS–polyacrylamide electrophoresis, transferred onto PVDF membranes, and incubated with specific antibodies against Enpp1 (1:1000; Bioss Antibodies Inc., Woburn, MA, USA), MKK3 (1:1000; ABclonal, China), phosphorus-p38 mitogen-activated protein kinase (MAPK) (1:1000; Servicebio, China), p38 MAPK (1:1000; ABclonal), PCNA (1:1000; ABclonal), and β-tubulin (1:1000; ABclonal). Membranes were then incubated with 1:1000 horseradish peroxidase-conjugated secondary antibody. Antibody-antigen complexes were visualized using an ECL reagent, and images were captured using the ImageQuant™ LAS 4000 imager (Fujifilm, Tokyo, Japan).

### Statistical analysis

All statistical analyses were conducted using the SPSS software (version 22.0; IBM SPSS, NY, USA). The data are expressed as the mean ± standard deviation. All data were analyzed using GraphPad Prism 6.02 (GraphPad Software Inc., San Diego, CA, USA). Comparisons between the two groups were performed using the Student’s t test, and comparisons between multiple groups were conducted using analysis of variance. Statistical significance was set at *P* < 0.05.

## Results

### Enpp1 deletion inhibited body weight gain and decreased trabecular bone mass

**Fig. 1:**
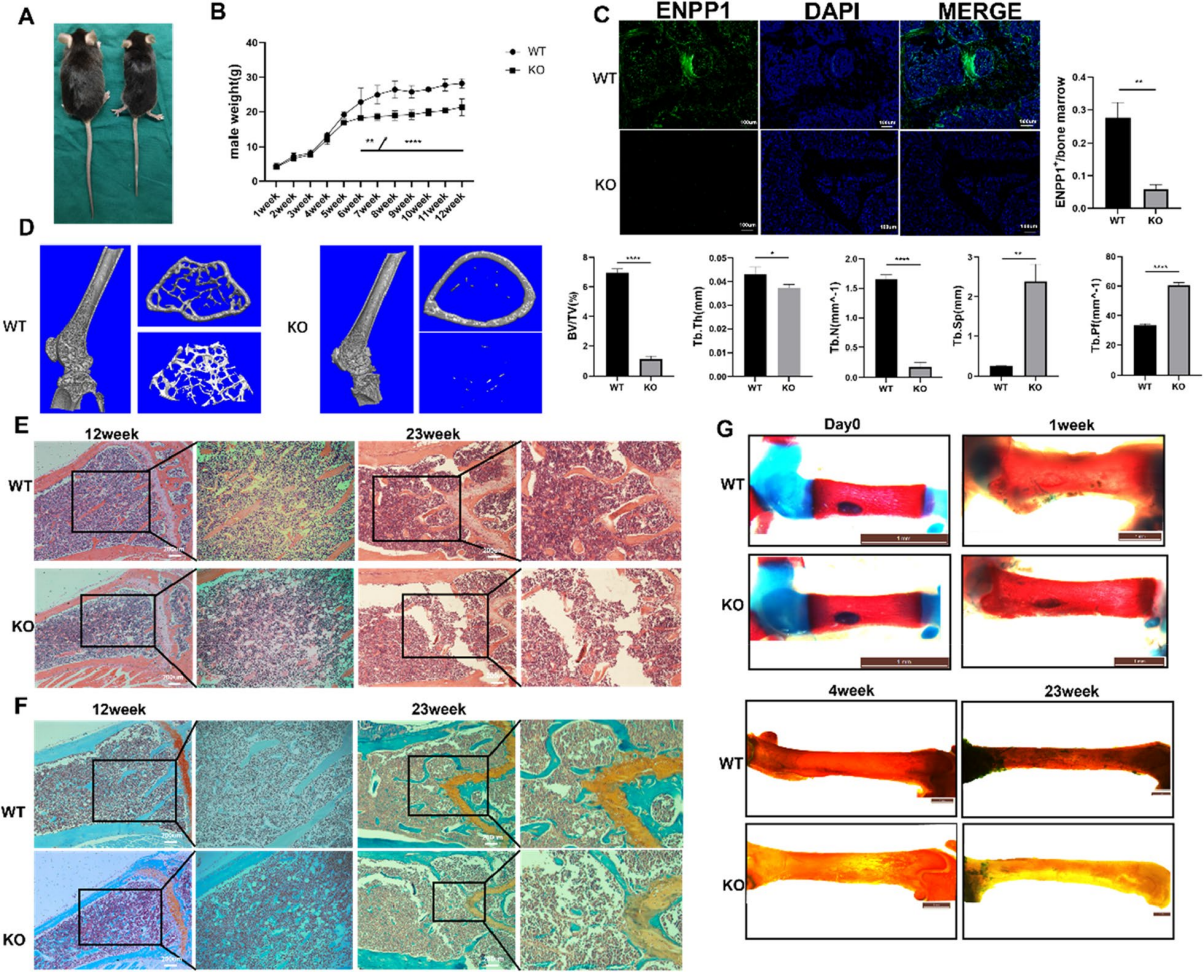
Histology and micro-computed tomography of trabecular bone in femur. **A** The body size of wild-type (WT) (left) and knockout (KO) (right) mice was compared after anesthesia at 12 weeks of age. **B** Body weights of 3–4 mice from each group were noted. **C** Immunofluorescence staining and quantification of the expression of Enpp1 (green) in mouse distal femur. Cell nuclei were stained blue using DAPI. Scale bar: 100 µm. **D** Representative μCT scans of the distal femur showed 3D reconstructed trabecular bones of WT and KO mice at 12 weeks of age. Quantitative analysis of the percentage of bone volume (BV/TV), trabecular thickness (Tb. Th), trabecular number (Tb. N), trabecular spacing (Tb. Sp), trabecular pattern factor (Tb. Pf) (*n* = 3) was conducted. **E** Hematoxylin and eosin staining and **F** saffron solid green staining images of femur near the metaphysic area of 12- and 23-week-old mice. The right panel shows a higher magnification(X100). **G** Alcian blue and Alizarin Red staining of femur of mice from birth to 23 weeks of age (cartilage elements and bones are labeled in blue and red, respectively). Densitometric analysis (mean + SD) from three independent experiments is presented

No significant difference in body weight was found between the mice in the WT and KO groups before 6 weeks of age, whereas the body weight of WT mice increased significantly compared with that of KO mice in the following weeks (Fig. 1B;  *p* < 0.001). At 12 weeks of age, the body weight of WT mice (28.20 ± 1.37 g, *n* = 4) was approximately 1.3 times that of the KO mice (21.30 ± 2.43 g, *n* = 3; *p* < 0.05). *Enpp1* KO mice (right) showed significant growth arrest (Fig. 1A) and reduced values of bone volume/total volume (BV/TV, 18.5% of the WT), trabecular thickness (Tb. Th, 86.0% of the WT), trabecular number (Tb. N, 22.5% of the WT), and significantly higher trabecular separation (Tb. Sp, 1.76 times of the WT), trabecular pattern factor (Tb. Pf, 1.80 times of the WT), and structure model index (SMI, 1.20 times of the WT) in the distal femur than WT mice (left). Cross-sectional CT reconstruction at approximately 1000um proximal to the femoral growth plate revealed significantly more sparse trabecular bone in KO mice (Fig. 1D; Table 1, all *p* < 0.05). At 23 weeks of age, *Enpp1* KO mice walked on the tiptoe, developed a severe decline in mobility, and showed body stiffness compared to WT mice (see Additional file 1: Video). Unlike WT mice, female *Enpp1* KO mice did not produce pups during the study. Immunofluorescence staining was performed at the same location near the growth plate, at the distal end of the femur, at 23 weeks of age. The WT group showed higher expression of *Enpp1* in the distal femurs, as revealed by the high integrated optical density (Fig. 1C, *p* = 0.0016). At the age of 12 weeks, HE and saffron solid green staining of the distal femur showed dense cancellous bone and few vacuoles in the WT group; however, trabecular bone was loosely arranged, with some vacuoles and a decrease in trabecular bone thickness in the KO group. Similarly, at the age of 23 weeks, sparser and thinner trabecular bone with more vacuoles in the medulla, worsened structural integrity, and increased trabecular bone separation was observed in the KO group compared to that in the WT group (Fig. 1E, F). For staining of the skeleton, cartilage elements and bones were labeled in blue and red, respectively. Less Alizarin Red staining of the femur was detected in KO mice compared to that in WT mice at 4 and 23 weeks of age, especially at 23 weeks of age (Fig. 1G).

**Table 1:** Trabecular bone parameters in the distal femur of 12-week WT and KO mice

	WT	KO	*p* value
*Parameters of µCT*			
BV/TV (%)	6.967 ± 0.256	1.287 ± 0.220	**< 0.0001**
Tb. Th(mm)	0.043 ± 0.003	0.037 ± 0.001	**0.0441**
Tb. N(mm^−1^)	1.651 ± 0.078	0.372 ± 0.020	**< 0.0001**
Tb. Sp(mm)	0.255 ± 0.009	0.449 ± 0.042	**0.0014**
Connectivity	231.333 ± 11.060	104.000 ± 14.422	**0.0003**
Tb. Pf(mm^−1^)	33.477 ± 0.950	60.404 ± 2.033	**< 0.0001**
SMI	2.259 ± 0.111	2.703 ± 0.123	**0.0096**

Parameters were expressed as mean ± standard deviation, and comparisons between the two groups were performed using the Student’s t test

*p* value with statistical significance was labeled in bold

### Enpp1 deletion causes reduced proliferation and osteogenesis and increased apoptosis of cells in bone

**Fig. 2:**
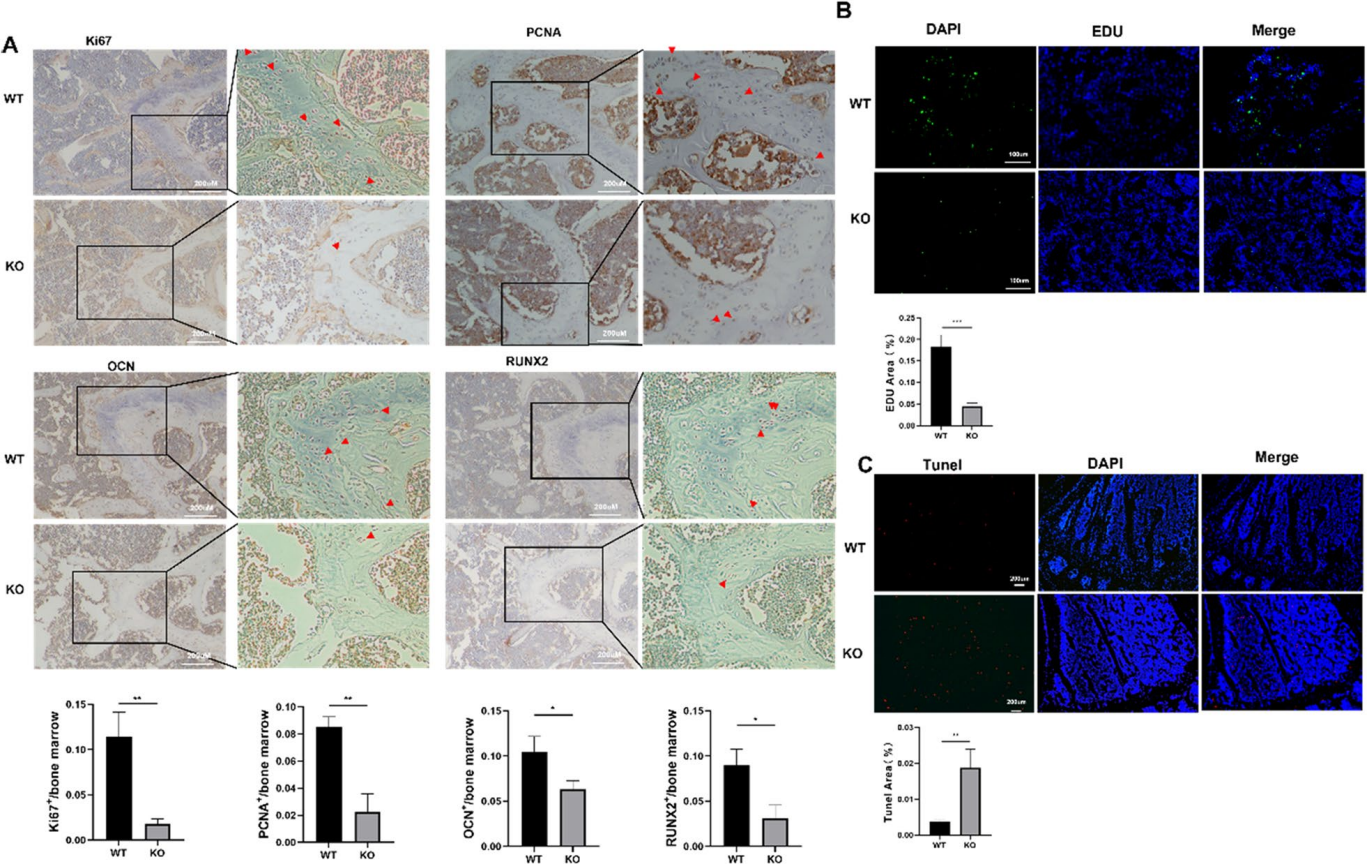
Histological proliferation, differentiation, and apoptosis of cells in bone **A** Immunohistochemical expression pattern of Ki67 (nuclear proliferation-associated antigen Ki67), PCNA (proliferating cell nuclear antigen), OCN (osteocalcin), and RUNX2(Runt-related transcription factor 2) in distal femurs of 23-week-old mice (partial positive signals are marked with red arrows) (*n* = 3). **B** EdU immunostaining and (**C**) TUNEL of proximal tibia in 12-week-old mice (*n* = 3)

Distal femur sections of 23-week-old mice were used for immunohistochemistry. The results demonstrated a significant increase in the levels of biomarkers for differentiation (OCN and RUNX2) and proliferation (Ki67 and PCNA) near the growth plate in WT mice compared to that in *Enpp1* KO mice (partial positive signals are marked with red arrows) (Fig. 2A, *p* < 0.05). During the characterization of cellular proliferation within the proximal tibia of 12-week-old mice after EdU intraperitoneal injection, we observed a significant reduction (75% decrease) in the percentage of EdU-positive cells in *Enpp1* KO mice compared to that in WT mice (Fig. 2B, *p* < 0.001). In contrast, TUNEL-positive cells increased in the proximal tibia of *Enpp1* KO mice as compared to WT mice (Fig. 2C, 80% increase, *p* = 0.0014). These observations indicated that *Enpp1* mutants inhibited proliferation and osteogenesis while promoting the apoptosis of cells in the bone.

### High-throughput protein sequencing shows downregulated MAPK signaling in the Enpp1 deleted bone

**Fig. 3:**
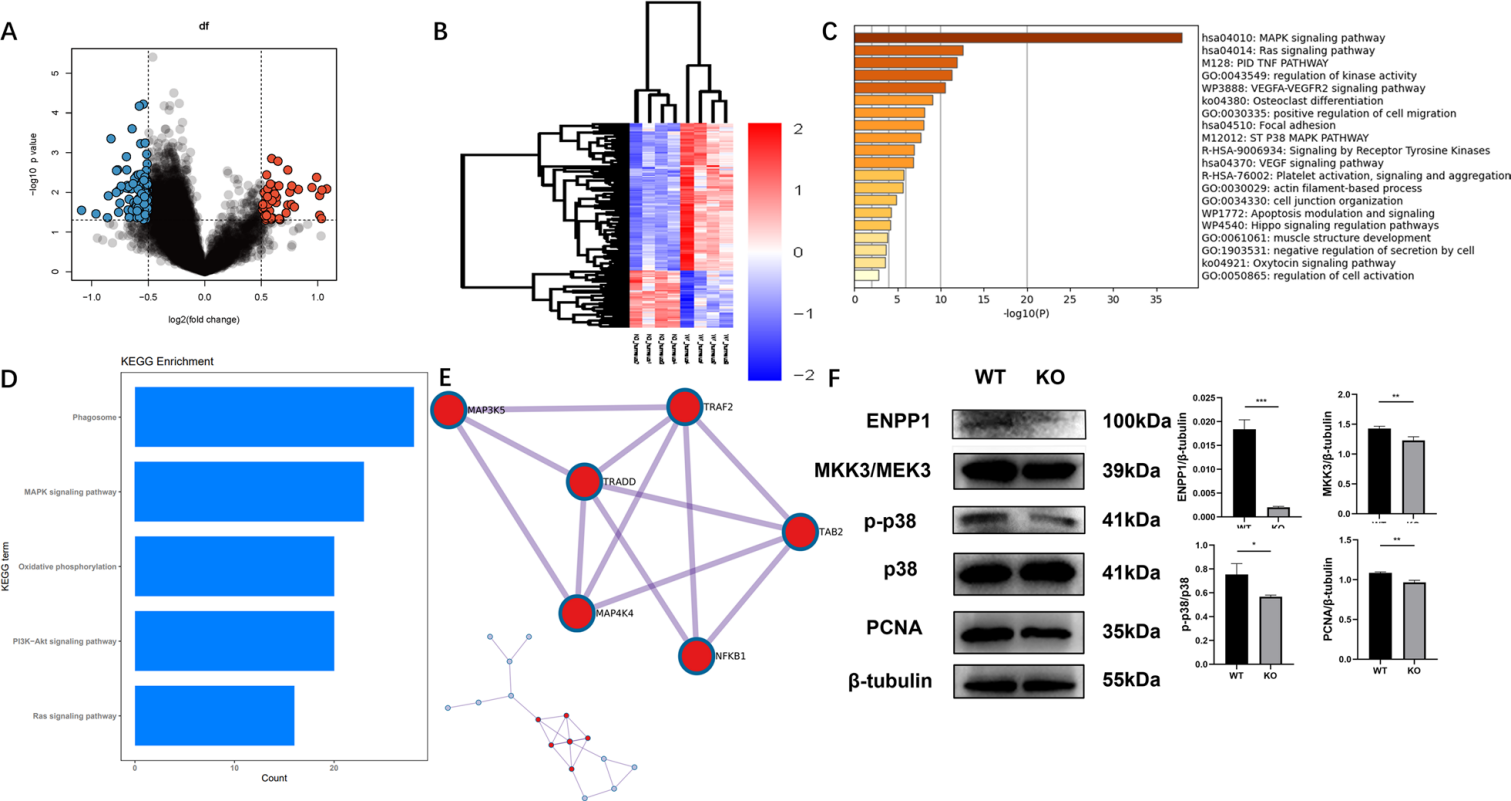
Downregulated expression of the MKK3/p38 MAPK/PCNA pathway in humeri from Enpp1 KO mice **A** Volcano plot shows proteins in the humeri of Enpp1 KO mice, which cross the significance threshold with displaying decreased 1268 proteins and increased 111 proteins in humeri of KO mice compared with that in WT mice. Enpp1 protein expression was significantly lower in KO than in the WT group. **B** Heatmap of 1379 differential expression proteins in the humeri of WT and Enpp1 KO mice (*p* < 0.005). Decreased expression in blue and increased expression in red. **C** Gene Ontology biological process analysis showed that differently expressed proteins are involved in the MAPK signaling and MAPK signaling pathway was significantly downregulated in Enpp1-deficient humeri when compared to that in the WT group (*p* = 10^−37.9533^). **D** Kyoto Encyclopedia of Genes and Genomes pathway analysis unveiled that the expression of the MAPK signaling pathway was downregulated in the humeri of Enpp1 knockout mice compared to that in the humeri of mice in the WT group. **E** Protein–protein interaction network diagram of key modules was related to MAP3K5. **F** Immunoblot result of MKK3, phosphorylated p38 MAPK, and PCNA of the humeri from WT and Enpp1 KO mice (*n* = 3). To improve clarity and conciseness, blots are cropped to the location of the target protein band

To identify the differentially expressed proteins in bone following *Enpp1* knockout, the deltoid and triceps muscles were dissected free from humeri and cleaned of excess connective tissue, and 1379 differentially expressed proteins in the humeri of WT and *Enpp1* KO mice were used to create a cross-sectional heatmap (Fig. 3B, *p* < 0.005).The distribution of differentially expressed proteins between KO and WT cohorts was visualized on a volcano plot, the result showed the proteins crossing the significance threshold with displaying decreased 1268 proteins and increased 111 proteins in humeri of KO mice compared with WT mice, and *Enpp1* expression was found to be significantly downregulated in KO mice compared to that in WT mice (Fig. 3A). GO biological process analysis showed that differently expressed proteins are involved in the MAPK signaling and MAPK signaling pathway was significantly downregulated in *Enpp1*-deficient humeri when compared to that in the WT group (Fig. 3C, *p* = 10^−37.9533^). KEGG pathway analysis also revealed that the MAPK signaling pathway was downregulated in the humeri of *Enpp1* KO mice (Fig. 3D). The top 20 clusters and their representative enriched terms (one per cluster) were derived (Table 2). MCODE cluster analysis tool was used to generate a correlated function network module. The interactions of proteins were visualized by the protein–protein interaction network diagram, and it revealed MAP3K5 as a possible signaling hub and validated the significantly expressed protein in humeri of 12-week-old mice that are related to MAP3K5 (Fig. 3E). To validate the proteomic results and EdU assay of bone, humeri-derived proteins of *Enpp1* KO mice showed MKK3/p38 MAPK/PCNA pathway-related proteins were significantly downregulated compared with WT mice, which was consistent with the results of high-throughput protein sequencing and EdU assay (Fig. 3F, all *p* < 0.05).

**Table 2:** The top 20 clusters and their representative enriched terms (one per cluster)

Description	Count	%	Log10(*P*)	Log10(*q*)
MAPK signaling pathway	20	86.96	− 37.95	− 33.89
Ras signaling pathway	9	39.13	− 12.52	− 8.76
PID TNF PATHWAY	6	26.09	− 11.87	− 8.29
Regulation of kinase activity	11	47.83	− 11.26	− 7.74
VEGFA-VEGFR2 signaling pathway	9	39.13	− 10.47	− 7.11
Osteoclast differentiation	6	26.09	− 9.09	− 6.05
Positive regulation of cell migration	8	34.78	− 8.08	− 5.17
Focal adhesion	6	26.09	− 7.98	− 5.12
ST P38 MAPK PATHWAY	4	17.39	− 7.66	− 4.9
Signaling by receptor tyrosine kinases	7	30.43	− 6.87	− 4.21
VEGF signaling pathway	4	17.39	− 6.83	− 4.19
Platelet activation, signaling, and aggregation	5	21.74	− 5.7	− 3.23
Actin filament-based process	7	30.43	− 5.63	− 3.18
Cell junction organization	6	26.09	− 4.81	− 2.5
Apoptosis modulation and signaling	3	13.04	− 4.23	− 2.08
Hippo signaling regulation pathways	3	13.04	− 4.15	− 2.02
Muscle structure development	5	21.74	− 3.86	− 1.79
Negative regulation of secretion by cell	3	13.04	− 3.64	− 1.61
Oxytocin signaling pathway	3	13.04	− 3.6	− 1.57
Regulation of cell activation	4	17.39	− 2.76	− 0.86

“Count” is the number of genes in the user-provided lists with membership in the given ontology term. “%” is the percentage of all the user-provided genes that are found in the given ontology term. (Only input genes with at least one ontology term annotation are included in the calculation.) “Log10(P)” is the p value in log base 10. “Log10(q)” is the multi-test adjusted *p* value in log base 10

### Stimulation of p38 MAPK signaling enhances the proliferation of pre-osteoblasts, whereas an agonist of p38 MAPK inhibits the enhanced pre-osteoblasts proliferation

**Fig. 4:**
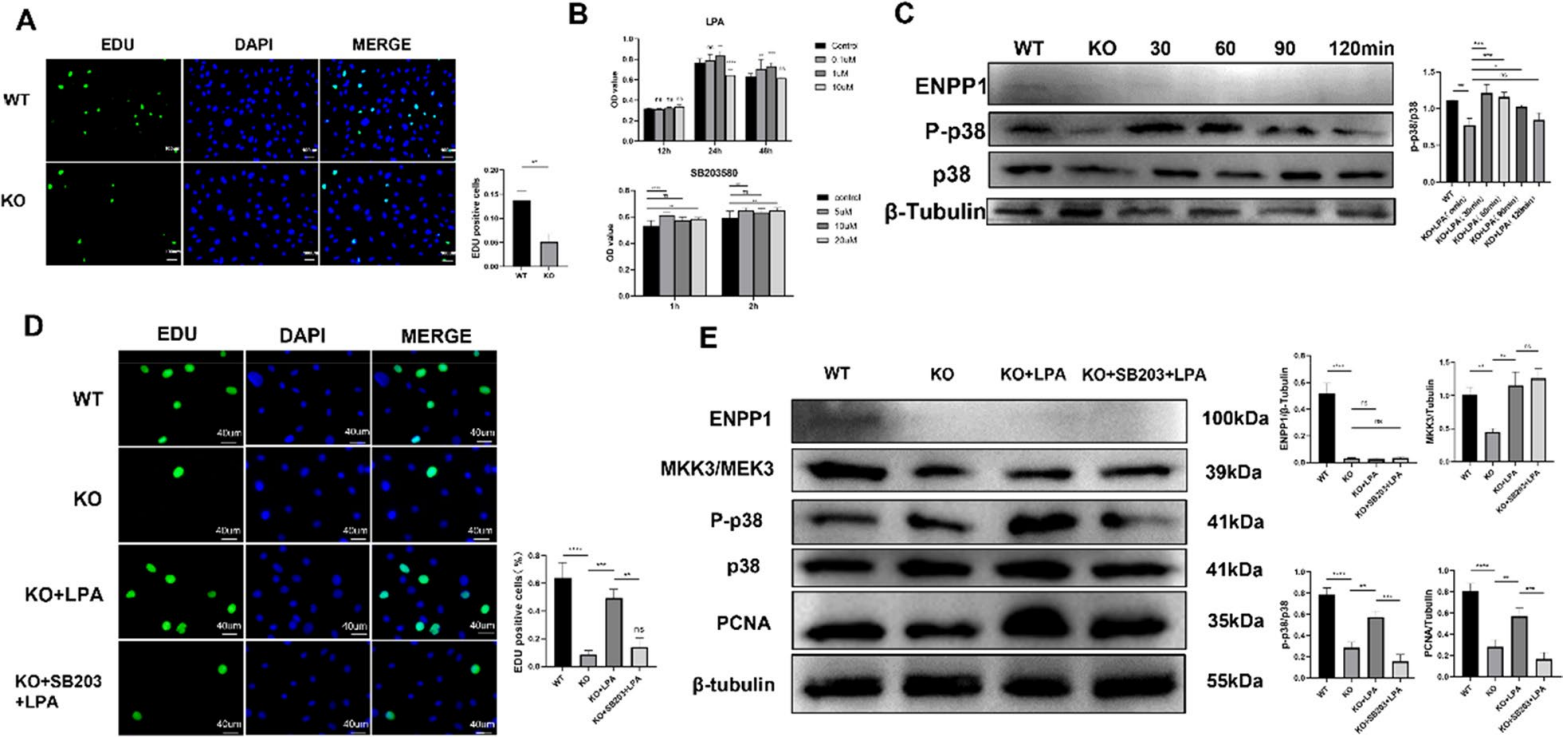
Lysophosphatidic acid (LPA) partially rescued the impacted proliferation of Enpp1 KO pre-osteoblasts via the MKK3/p38 MAPK/PCNA pathway **A** EdU immunostaining of Pobs from WT and Enpp1 KO suckling mice. EdU-positive cells were quantified (*n* = 3). **B** Cell viability measurement of Pobs incubated with LPA under a series of concentrations (0–10 μM) and times (12–48 h) or incubated with SB203580 (0–20 µM, 1 h, and 2 h) (*n* = 8). **C** Immunoblot showed the expression of phosphorylated p38 MAPK/p38 MAPK of WT and Enpp1 KO Pobs at different time gradients (0, 30, 60, 90, and 120 min) under LPA stimulation at 1 uM concentration (*n* = 3). **D** Pobs of WT and Enpp1 KO groups were incubated in the presence of LPA (1 μM) or SB203580 (10 μM; added 2 h prior to LPA addition) followed by LPA (1 µM) for 48 h. Cells were then stained for EdU immunostaining and visualized using confocal microscopy. Fluorescence intensity was quantified using Image-Pro Plus 6.0 (Media Cybernetics). Three different areas per chamber were measured. **E** Serum-starved Pobs were treated with LPA in the absence or presence of SB203580 (10 μM) that was added 2 h prior to LPA addition. MKK3/p38 MAPK/PCNA pathway expression was monitored using western blot. One representative blot for each protein and the densitometric analysis (mean + SD) from three independent experiments are presented. To improve clarity and conciseness, blots are cropped to the location of the target protein band. (Results are presented as the mean ± SD, *p < 0.05, **p < 0.01, ***p < 0.001; one-way analysis of variance with Bonferroni correction)

During cell culture, it was found that the proliferation ability of Pobs of KO suckling mice was evidently weak, which was confirmed by the EdU assay (Fig. 4A, *p* < 0.01). This is also consistent with the low expression of humeri-derived proliferation-related proteins in *Enpp1*-deficient humeri mentioned above. Pobs were subjected to lysophosphatidic acid (LPA) and the p38 MAPK selective inhibitor, pyridinyl imidazole molecule SB203580. The CCK-8 assay was used to evaluate cell viability over a range of time points and drug concentrations. When we administered 10 µM SB203580 for 2 h and 1 µM LPA for 48 h, the drugs showed no toxicity on cell activity (Fig. 4B). To verify the phosphorylation of p38 MAPK by LPA, five different LPA processing times (0, 30, 60, 90, and 120 min; 1 µM) were tested. Treatment from 0 to 90 min upregulated the expression of phosphorus-p38 MAPK compared to that in the untreated group (Fig. 4C, all *p* < 0.05). Therefore, this concentration (1 µM) was used in the subsequent experiments. After treatment with LPA (1 µM) for 48 h, the proliferation of Pobs in the KO group increased (*p* = 0.0006), whereas the promotion of proliferation was inhibited when Pobs were pretreated with SB203580 for 2 h (Fig. 4D, *p* = 0.0015). Similarly, the protein expression of MKK3/phosphorus-p38 MAPK/PCNA increased after LPA treatment, whereas SB203580 inhibited the expression of phosphorus-p38 MAPK and PCNA (Fig. 4E, all *p* < 0.05) (Additional file 2: Original western blot).

## Discussion

The skeletal and biochemical phenotypes of *Enpp1*-deficient humans and homozygous-deficient mice are nearly identical, demonstrating diminished skeletal bone mass and abnormally low levels of PPi [16, 17]. In this study, male *Enpp1* KO mice were confirmed to be an animal model of early-onset osteoporosis through phenotypic trait assessment, including reduced body weight and lower bone mass. To explore the mechanism of early-onset osteoporosis induced by *Enpp1* deficiency, we employed proteomics to analyze the protein profile of humeri from 12-week-old male WT and *Enpp1* KO mice. Proteomic analysis showed that strong genetic signals were associated with the MAPK signaling pathway and MAP3K5 (a key member of the p38 MAPK pathway), which were significantly downregulated in *Enpp1*-deficient bone, whereas the expression of JNK and ERK/MAPK signaling pathways was not significantly downregulated as that of the p38 MAPK signaling pathway (not shown). The p38 MAPK signaling pathway is implicated in a series of biological processes in which extracellular stimuli are transduced into different cellular responses. Once the stimuli reach the cell, MAP3K5 is activated and phosphorylates MKK3, which in turn phosphorylates and activates p38 MAPK [18]. The MKK3/p38 MAPK axis is required to regulate pre-osteoblast genesis programs [19–21]. For p38 MAPK inhibitor (SB203580) impaired MC3T3 pre-osteoblast differentiation [22] and the role of MAP3K in the activation of p38 MAPK [23]. It is worth confirming that the MAP3K5–MKK3–p38 MAPK signaling pathway responds to *Enpp1* deficiency and is regulated by *Enpp1*. This was verified by detecting protein expression in the humeri in our study. This outcome opens the possibility of changes in bone structure because of changes in p38 MAPK signaling pathway expression. In addition, we observed significantly inhibited proliferation of Pobs in the *Enpp1* KO group compared to that in the WT group during cell culture. Considering the role of *Enpp1* deficiency in the suppression of pre-osteoblast proliferation and important role of p38 MAPK in cell proliferation [24, 25], it is possible that the p38 MAPK signaling pathway plays a vital role in the decreased proliferation of Pobs caused by *Enpp1* deletion. To prove our hypothesis, lysophosphatidic acid (LPA) was used in our study, which has been already demonstrated in induction of pre-osteoblast proliferation in MC3T3-E1 cells [26, 27]. However, there have been no prior studies on the effect of LPA on the p38 MAPK pathway in pre-osteoblasts. In our study, we found that LPA activated the expression of the MKK3/p38 MAPK/PCNA pathway and promoted the proliferation of pre-osteoblasts, indicating that the inhibition of pre-osteoblast proliferation caused by *Enpp1* deletion could be reversed by upregulation of the MKK3/p38 MAPK/PCNA pathway, and it can be inhibited by a selective p38 MAPK inhibitor (SB203580).

However, a limitation of this study is that there are differences in the pre-osteoblast microenvironment in vivo and in vitro, and the therapeutic effect of LPA in vivo is unclear. In addition, tissue proliferation and apoptosis staining were not concentrated on the surface of trabecular bone but all over the bone marrow cavity of the long bone, indicating that the proliferation and apoptosis of bone marrow mesenchymal stem cells were also affected by *Enpp1* knockout, which needs to be further verified. In conclusion, our findings suggested that *Enpp1* deficiency-associated osteoporosis involves inhibition of the MKK3/p38 MAPK/PCNA signaling pathway and that LPA partially rescued the impacted proliferation of pre-osteoblasts via the MKK3/p38 MAPK/PCNA pathway, which deepened our understanding of the mechanism of early-onset osteoporosis caused by deletion mutation of *Enpp1* gene and provides some help for clinical treatment.

## Supplementary Information

**Additional file 1:** Walking gaits of 23-week-old WT and ENPP1 KO mice.

**Additional file 2:** Original western blot.

## Data Availability

Authors can confirm that all relevant data are included in the article or its supplementary information files.

## Abbreviations

ENPP1: Ectonucleotide pyrophosphatase/phosphodiesterase 1
MAPK: Mitogen-activated protein kinase
MKK3: MAP Kinase Kinase3
PCNA: Proliferating cell nuclear Antigen
PPi: Pyrophosphate
LPA: Lysophosphatidic acid
Pob: Calvarial pre-osteoblast

## References

[1] Huang R, Rosenbach M, Vaughn R, Provvedini D, Rebbe N, Hickman S (1994). Expression of the murine plasma cell nucleotide pyrophosphohydrolase PC-1 is shared by human liver, bone, and cartilage cells: regulation of PC-1 expression in osteosarcoma cells by transforming growth factor-beta. J Clin Invest..

[2] Liang J, Fu M, Ciociola E, Chandalia M, Abate N (2007). Role of ENPP1 on adipocyte maturation. PLoS ONE.

[3] Fiona Roberts DZ, Farquharson C, Vicky EM (2019). ENPP1 in the regulation of mineralization and beyond. Trends Biochem Sci.

[4] Bollen M, Gijsbers R, Ceulemans H, Stalmans W, Stefan C (2000). Nucleotide pyrophosphatases/phosphodiesterases on the move. Crit Rev Biochem Mol Biol.

[5] Oheim RZK, Maulding ND, Stürznickel J, von Kroge S, Kavanagh D, Stabach PR, Kornak U, Tommasini SM, Horowitz MC, Amling M, Thompson D, Schinke T, Busse B, Carpenter TO, Braddock DT (2020). Human heterozygous ENPP1 deficiency is associated with early onset osteoporosis, a phenotype recapitulated in a mouse model of Enpp1 deficiency. J Bone Miner Res.

[6] Okawa A, Nakamura I, Goto S, Moriya H, Nakamura Y, Ikegawa S (1998). Mutation in Npps in a mouse model of ossification of the posterior longitudinal ligament of the spine. Nat Genet.

[7] Harmey D, Hessle L, Narisawa S, Johnson KA, Terkeltaub R, Millán JL (2004). Concerted regulation of inorganic pyrophosphate and osteopontin by akp2, enpp1, and ank: an integrated model of the pathogenesis of mineralization disorders. Am J Pathol.

[8] Shull LC, Sen R, Menzel J, Goyama S, Kurokawa M, Artinger KB (2020). The conserved and divergent roles of Prdm3 and Prdm16 in zebrafish and mouse craniofacial development. Dev Biol.

[9] Buo AM, Tomlinson RE, Eidelman ER, Chason M, Stains JP (2017). Connexin43 and Runx2 interact to affect cortical bone geometry, skeletal development, and osteoblast and osteoclast function. J Bone Miner Res.

[10] Yang L, Liu J, Shan Q, Geng G, Shao P (2020). High glucose inhibits proliferation and differentiation of osteoblast in alveolar bone by inducing pyroptosis. Biochem Biophys Res Commun.

[11] Hu S, Zhang C, Ni L, Huang C, Chen D, Shi K (2020). Stabilization of HIF-1α alleviates osteoarthritis via enhancing mitophagy. Cell Death Dis.

[12] Willemsen M, Krebbers G, Bekkenk MW, Teunissen MBM, Luiten RM (2021). Improvement of opal multiplex immunofluorescence workflow for human tissue sections. J Histochem Cytochem.

[13] Markaryan A, Nelson EG, Tretiakova M, Hinojosa R (2008). Technical report: immunofluorescence and TUNEL staining of celloidin embedded human temporal bone tissues. Hear Res.

[14] Chan RW, Gargett CE (2006). Identification of label-retaining cells in mouse endometrium. Stem Cells.

[15] Yang HY, Kwon J, Kook MS, Kang SS, Kim SE, Sohn S (2013). Proteomic analysis of gingival tissue and alveolar bone during alveolar bone healing. Mol Cell Proteomics.

[16] Babij P, Roudier M, Graves T, Han CY, Chhoa M, Li CM (2009). New variants in the Enpp1 and Ptpn6 genes cause low BMD, crystal-related arthropathy, and vascular calcification. J Bone Miner Res.

[17] Mackenzie NC, Zhu D, Milne EM, vant Hof R, Martin A, Darryl Quarles L, et al. Altered bone development and an increase in FGF-23 expression in Enpp1(-/-) mice. PLoS One. 2012;7(2):e32177. 10.1371/journal.pone.0032177 PMC3281127 22359666

[18] Rodríguez-Carballo E, Gámez B, Ventura F (2016). p38 MAPK signaling in osteoblast differentiation. Front Cell Dev Biol.

[19] Greenblatt MB, Shim JH, Zou W, Sitara D, Schweitzer M, Hu D (2010). The p38 MAPK pathway is essential for skeletogenesis and bone homeostasis in mice. J Clin Invest.

[20] Xie B, Zeng Z, Liao S, Zhou C, Wu L, Xu D (2021). Kaempferol ameliorates the inhibitory activity of dexamethasone in the osteogenesis of MC3T3-E1 cells by JNK and p38-MAPK pathways. Front Pharmacol.

[21] Tachi J, Tokuda H, Onuma T, Yamaguchi S, Kim W, Hioki T (2020). Duloxetine strengthens osteoblast activation by prostaglandin E(1): upregulation of p38 MAP kinase. Prostaglandins Other Lipid Mediat.

[22] Suzuki A, Guicheux J, Palmer G, Miura Y, Oiso Y, Bonjour JP (2002). Evidence for a role of p38 MAP kinase in expression of alkaline phosphatase during osteoblastic cell differentiation. Bone.

[23] Cuadrado A, Nebreda AR (2010). Mechanisms and functions of p38 MAPK signalling. Biochem J.

[24] Zhu L, Lin ZW, Wang G, Zhang H, Liu B, Xu QJ (2019). MicroRNA-495 downregulates AQP1 and facilitates proliferation and differentiation of osteoblasts in mice with tibial fracture through activation of p38 MAPK signaling pathway. Sci Rep.

[25] Liu M, Fan F, Shi P, Tu M, Yu C, Yu C (2018). Lactoferrin promotes MC3T3-E1 osteoblast cells proliferation via MAPK signaling pathways. Int J Biol Macromol.

[26] Masiello LM, Fotos JS, Galileo DS, Karin NJ (2006). Lysophosphatidic acid induces chemotaxis in MC3T3-E1 osteoblastic cells. Bone.

[27] Yu ZL, Li DQ, Huang XY, Xing X, Yu RQ, Li Z (2016). Lysophosphatidic acid upregulates connective tissue growth factor expression in osteoblasts through the GPCR/PKC and PKA pathways. Int J Mol Med.

