# Supplementary material for: ENPP1 deletion causes mouse osteoporosis via the MKK3/p38 MAPK/PCNA signaling pathway
Source: J Orthop Surg Res. 2022 Oct 15;17:455. doi: 10.1186/s13018-022-03349-1 (PMC9571425; doi:10.1186/s13018-022-03349-1)

Fig.3F

first

[illegible]-Tubulin1
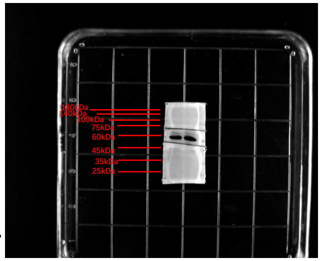

P-p38
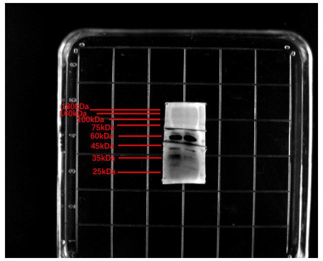

[illegible]-Tubulin2
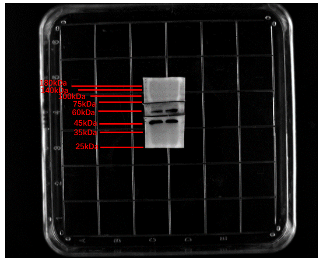

p38
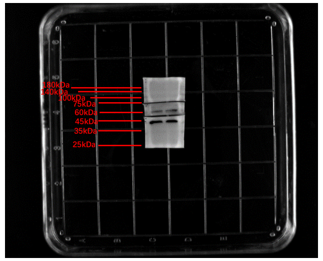

PCNA
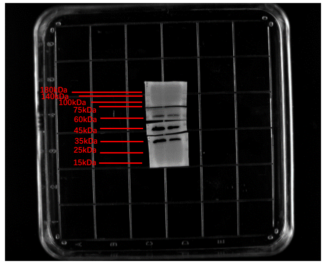

[illegible]-Tubulin3
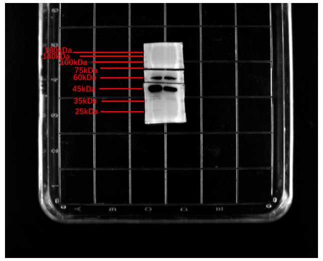

MKK3
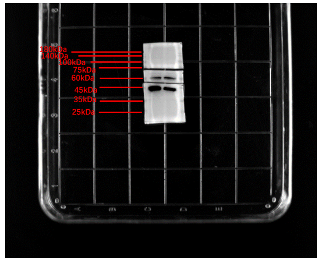

ENPP1
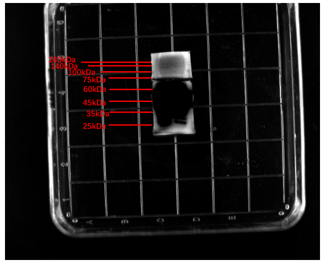

Fig.3F

second

Enpp1

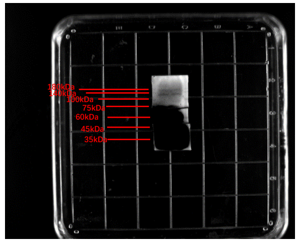

MKK3

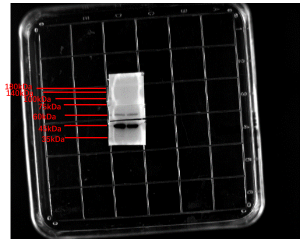

p38

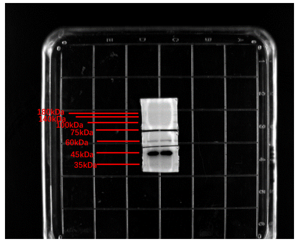

P-p38/
β-Tubulin

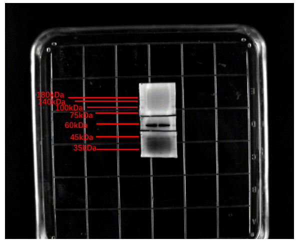

PCNA

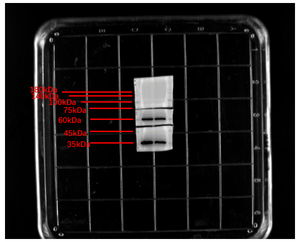

β-Tubulin

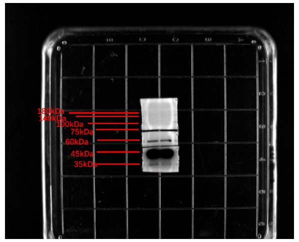

Fig.4C

first

ENPP1

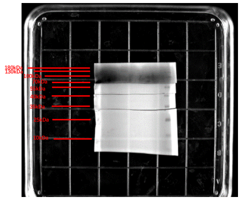

βtubulin

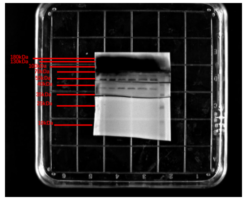

P-p38

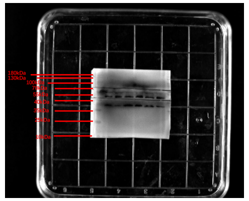

p38

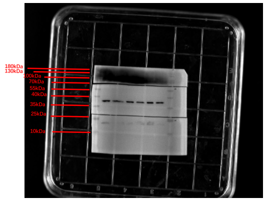

Fig.4C

second

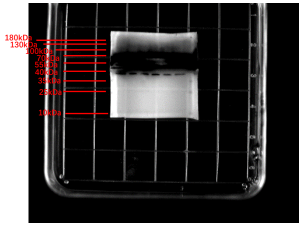

ENPP1

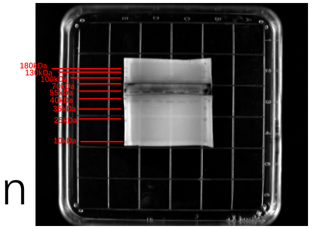

βtubulin

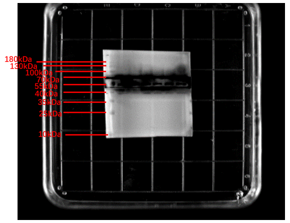

P-p38

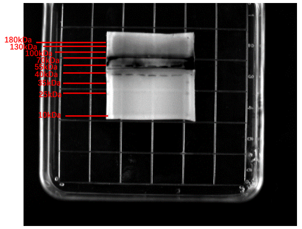

p38

Fig.4C

third

βtubulin

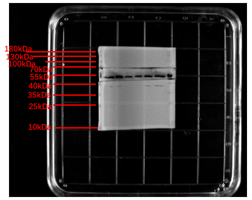

P-p38

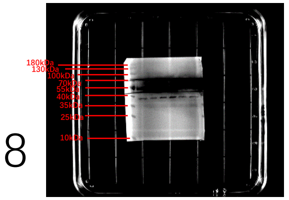

p38

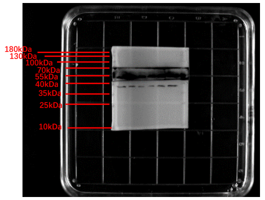

Fig.4E

first

ENPP1

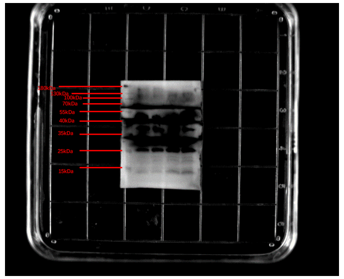

MKK3

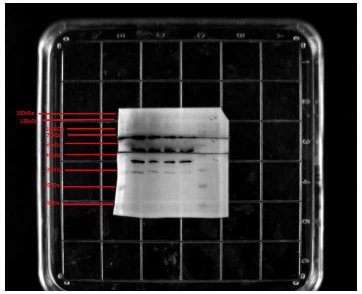

P-p38

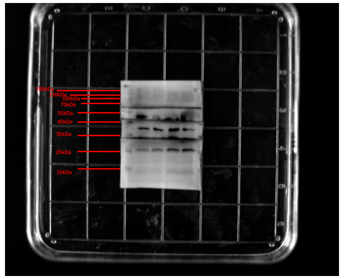

p38

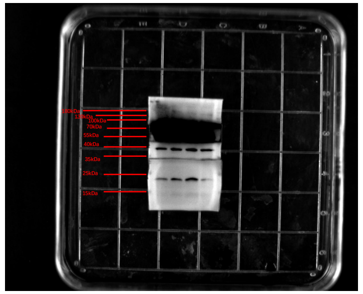

PCNA

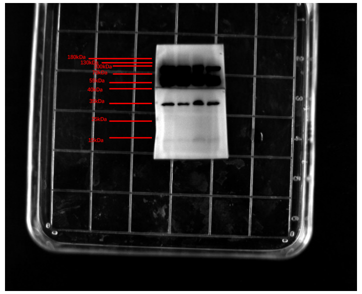

βtubulin

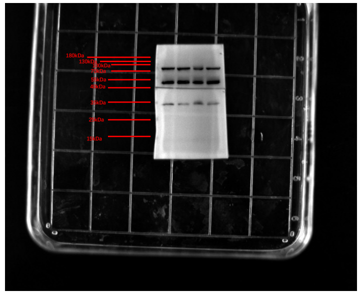

Fig.4E

second

ENPP1

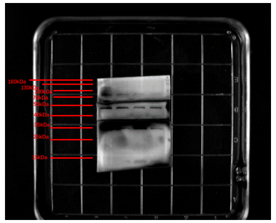

MKK3

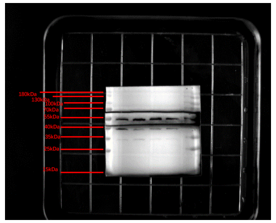

P-p38

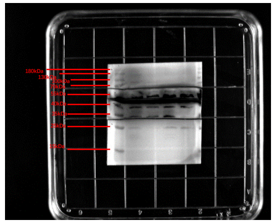

p38

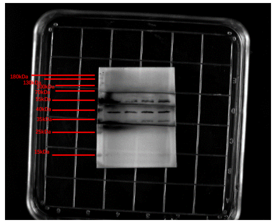

PCNA

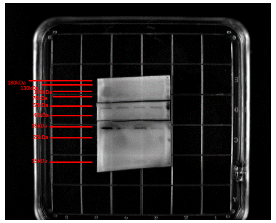

βtubulin

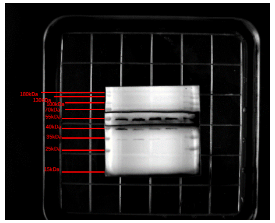

Fig.4E

third

ENPP1

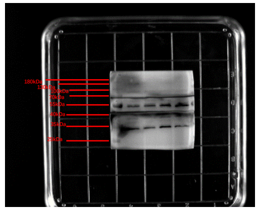

MKK3

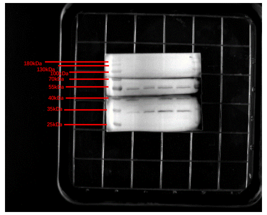

P-p38

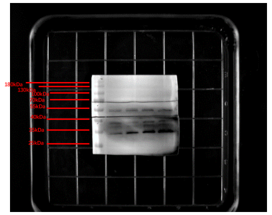

p38

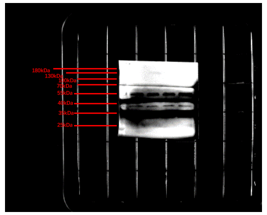

PCNA

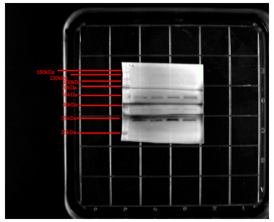

βtubulin

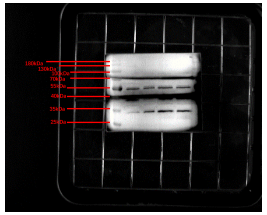

Supplement: Supplementary file 2 — Additional file 2: Original western blot. [file 13018_2022_3349_MOESM2_ESM.pdf]
